# Comprehensive analysis of lncRNA-mediated ceRNA networkfor hepatocellular carcinoma

**DOI:** 10.3389/fonc.2022.1042928

**Published:** 2022-10-21

**Authors:** Weiqing Chen, Feihua Chen, Mouchun Gong, Zhaoqing Jin, Lilu Shu, Zhi-wei Wang, Jianjiang Wang

**Affiliations:** ^1^ First People’s Hospital of Hangzhou Lin’an District, Affiliated Lin’an People’s Hospital, Hangzhou Medical College, Hangzhou, China; ^2^ Department of Research and Development, Zhejiang Zhongwei Medical Research Center, Hangzhou, China; ^3^ Department of Obstetrics and Gynecology, The Second Affiliated Hospital of Wenzhou Medical University, Wenzhou, China

**Keywords:** HCC, lncRNA, HOTTIP, MiR-205, prognosis, immune microenvironment

## Abstract

**Background:**

Hepatocellular carcinoma (HCC) is a high-burden cancer. The molecular mechanism of HCC has not been fully elucidated. Notably, current research has revealed a significant function for long non-coding RNAs (lncRNAs) in the prognosis of patients with HCC. Here, this study aims to construct a regulated lncRNA-mediated ceRNA network and find biological targets for the treatment of HCC.

**Methods:**

Based on the RNA expression patterns from the TCGA, we did an analysis to determine which genes were expressed differently between liver tumor tissues and noncancerous tissues. Then, using bioinformatic tools, we built a lncRNA-miRNA-mRNA ceRNA network and used GO and KEGG functional analyses on the DEmRNAs connected to ceRNA networks. The main lncRNAs in the subnetwork were chosen, and we next looked at the relationships between these lncRNAs and the clinical characteristics of patients with HCC. The prognosis-related genes and immune cells were identified using Kaplan-Meier and Cox proportional hazard analyses, and CIBERSORT was utilized to separate the 22 immune cell types. CCK8 assay was performed to measure cell viability in HCC cells after lncRNA HOTTIP modulation.

**Results:**

Differentially expressed mRNA and lncRNAs in HCC and paracancerous tissues were identified. There are 245 lncRNAs, 126 miRNAs, and 1980 mRNAs that are expressed differently in liver tumour tissues than in noncancerous cells. Function analysis showed that mRNAs in ceRNA network were significantly enriched in G1/S transition of mototiv cell cycle, positive regulation of cell cycle process, hepatocellular carcinoma, and cancer related pathways. CD8 T cells and T follicular helper cells had a favourable link with a 0.65 correlation coefficient. Additionally, there was a strong correlation between Eosinophils, activated NK cells, and B memory cells. Strikingly, depletion of lncRNA HOTTIP inhibited viability of HCC cells. In addition, miR-205 upregulation suppressed viability of HCC cells, while miR-205 downregulation repressed viability of HCC cells. Notably, miR-205 depletion rescued HOTTIP depletion-mediated suppression of cell viability in HCC.

**Conclusion:**

A ceRNA network was created by examining the lncRNA, miRNA, and mRNA expression profiles of liver tumours from the TCGA database. LncRNA HOTTIP promoted cell viability *via* inhibition of miR-205 in HCC cells.

## Introduction

Hepatocellular carcinoma (HCC) is one of the most common tumors in the world (1). The epidemiology of HCC shows two main patterns: one in North America and Western Europe, and the other in non-Western countries, such as sub-Saharan Africa, Central and South-east Asia, and the Amazon Basin (2). Men are generally more affected than women, and most commonly between the ages of 30 and 50, and HCC causes 662,000 deaths worldwide each year, about half of them in China (3). By 2022, there will be 41,260 new cases and 30,520 new deaths according to cancer statistics (4).

The treatment of HCC can be divided into surgical treatment and non-surgical treatment (5). Surgical treatment includes resection, cryoablation and liver transplantation. Nonsurgical treatments include either liver-specific therapy (i.e., percutaneous ethanol injection, radiofrequency/microwave ablation, trans-arterial embolization, external radiation therapy) or systemic therapy (chemotherapy, molecular targeted therapy, immune checkpoint inhibitor therapy) (6). Surgical treatment is the main treatment for HCC, but the 5-year survival rate after surgical resection is only about 30% (7). Currently, the molecular mechanism of HCC occurrence has not been fully elucidated (8), so it is of great significance to explore the molecular mechanism of HCC occurrence and development and screen biological markers for the early diagnosis of HCC for the treatment of this deadly disease.

Long non-coding RNA (lncRNA) is a class of non-coding RNA with a length greater than 200bp, which has no protein-coding function, poor conserved between species, and strong tissue specificity and spatio-temporal specificity (9). Recently, more and more lncRNAs have been found to act as oncogenic or tumor suppressor factors, and many oncogenic genomic loci are mainly transcribed from those lncRNAs that play an important role in cancer induction (10–13). With the rapid development of cancer research, a large number of lncRNAs related to tumorigenesis have been identified (14–16). Studies have shown that lncRNA MEG3 can promote the proliferation of HCC cells (17, 18). At the same time, some studies have proved that lncRNAs HOTTIP and UCA1 can promote the deterioration of HCC. Epigenetic related molecules (such as SNRPC), noncoding RNAs (such as HSA-Mir-221) and immune-related molecules (such as DCK) can be used as potential biomarkers for diagnosis, treatment and prognosis of HCC (19–22).

Studies have shown that lncRNAs have a wide range of biological functions, such as participating in RNA generation and processing, transcription regulation and chromatin remodeling (23). However, the mechanism of action is complex, such as participating in transcriptional regulation by binding characteristic proteins or taking part in post-transcriptional regulation as ceRNA (24). According to the ceRNA hypothesis proposed in 2011, when circRNA and mRNA share the same miRNA response element (MRE), they will competitively bind MRE to regulate the expression level of related genes. The most common mechanism of action between lncRNAs and miRNAs is that lncRNAs directly complement and pair with miRNA seed sequences, adsorb miRNAs to form complexes, and reduce the number of miRNAs involved in downstream gene regulation in cells (25). Studies have shown that the abnormal expression of lncRNAs affects the expression of mRNAs through sponge miRNAs, leading to tumorigenesis and cancer progression (26, 27). Therefore, in order to better study the functions of lncRNAs, there is an urgent need to better understand the regulatory networks of lncRNAs. This article aimed to perform a comprehensive analysis of TCGA transcriptome data to identify differentially expressed lncRNAs, miRNAs, and mRNAs to generate a regulated ceRNA network.

## Methods:

### Database

Data were downloaded from the TCGA database (https://portal.gdc.cancer.gov/) (28). The data were used to study the differential expression of lncRNA, mRNA, miRNA among HCC patients. In addition, edgeR(3.28.0) (29) of R language was mainly used to identify differentially expressed RNA. Screening criteria is: |logFC| > 2, and *P* value < 0.01 for mRNA and miRNA, and |logFC| > 4, and *P* value < 0.01 for lncRNA.

### Construction of ceRNA network and prognosis model

Using the mircode database, the correlation analysis of differentially expressed lncRNAs and mRNAs was carried out. Then, a ceRNA network was built using the miRDB, miRTarBase, and TargetScan databases. By adding edge and nodal gene information to the interaction network with Cytoscape 3.8.0, the HCC ceRNA network was displayed. The Kyoto Encyclopedia of Genes and Genomes and Gene Ontology (GO) (30) were then used to annotate the differentially expressed mRNAs in the ceRNA network. The R language’s “clusterProfiler,” (31) “org.Hs.eg.db,” “enrichplot,” and “ggplot2” (32) packages were used to carry out the aforementioned analysis. According to the median model risk score, all patients were separated into high-risk and low-risk groups, and Kaplan-Meier survival curves were created to analyze the variations in survival between the two groups (33). It can be regarded as statistically significant when *P <*0.05. Using the area under the curve (AUC), the model’s predictive effectiveness was assessed (visualized by the “timeROC” package in R language). After that, the “rms” package of R was used to build the nomogram survival prediction map, and the calibration curve was used to assess the accuracy of the map in predicting the survival rate of HCC patients.

### Assessing immune cells

Using the “CIBERSORT” software tool in the R language, the abundance of 22 immune cell subsets in 424 tumour specimens were estimated (34). The threshold used was *P <*0.05. The “Pheatmap” programme was used in the study to show immune cell infiltration in HCC. To examine the immune cells that are connected to survival, we employed univariate Cox regression (35). Lasso regression and multivariate Cox regression analysis were used together to create the prognosis related model (36), and the model’s accuracy was assessed using the AUC value. We estimated the prognostic model’s quantification using the multi-index AUC value, and we determined the nomogram’s predictive power using the calibration curve. To investigate the connection between important genes in the ceRNA network and immune cells in the model, we employed Pearson correlation analysis (37).

### Construction of nomogram model

For the derived independent prognostic indicators, a nomogram model was created and shown. The concordance index (C-index) of the independent prognostic factors and the Nomogram model, comprised of risk group fitting with the Coxph model, were determined to validate the nomogram’s prediction abilities. The statistical test was then run with resampling, and the significant *P* < value was determined. The degree to which the independent prognostic factors and compound factors fitted the Coxph model was evaluated, and the factor with the lowest *P* value was chosen to draw the calibration curve. The model created by this component performed better when the calibration curve was closer to 45°.

### Transfection

The HOTTIP siRNA and negative control siRNA (NC), miR-205 mimics and miR-205 inhibitors were obtained from GenePharm (Shanghai, China). The HOTTIP and miR-205 were transfected into HCC cells by lipofectamine 3000 (Invitrogen) based on the manufacturer’s protocol.

### Reverse transcription-quantitative PCR analysis

Total RNA was isolated from HCC cell lines using TRIzol reagent based on the manufacturer’s protocol. RT-PCR was performed as described before. The primers are as follows: HOTTIP: forward primer: 5’-CCT AAA GCC ACG CTT CTT TG-3’; reverse primer: 5’-TGC AGG CTG GAG ATC CTA CT-3’; GAPDH: forward primer: 5’-GTC AAC GGA TTT GGT CTG TATT-3’; reverse primer: 5’-AGT CTT CTG GGT GGC AGT GAT-3’.

### Cell counting assay Kit-8 assay

Huh-7 and HepG2 HCC cell lines were cultured in Dulbecco’s minimal essential medium (DMEM) supplemented with 5% fetal bovine serum, 100 Units/ml penicillin and 100 μg/ml streptomycin, at 37°C under a 5% CO_2_ atmosphere. CCK-8 assay was performed to analyze cell proliferation based on the manufacture’s protocol in HCC cell lines after 48 and 72 h transfection. The OD450nm was measured by a microplate reader.

### Statistical analysis

Statistical analyses for CCK-8 assay were performed by GraphPad Prism 5.0 (Graph Pad Software, La Jolla, CA). The results were presented as mean ± standard deviation (SD). Multiple groups were analyzed by one-way analysis of variance (ANOVA) test followed Turkey’s posttest. *P* < 0.05 was indicated statistically significant.

## Results:

### Expression of DERNAs

**Figure 1:**
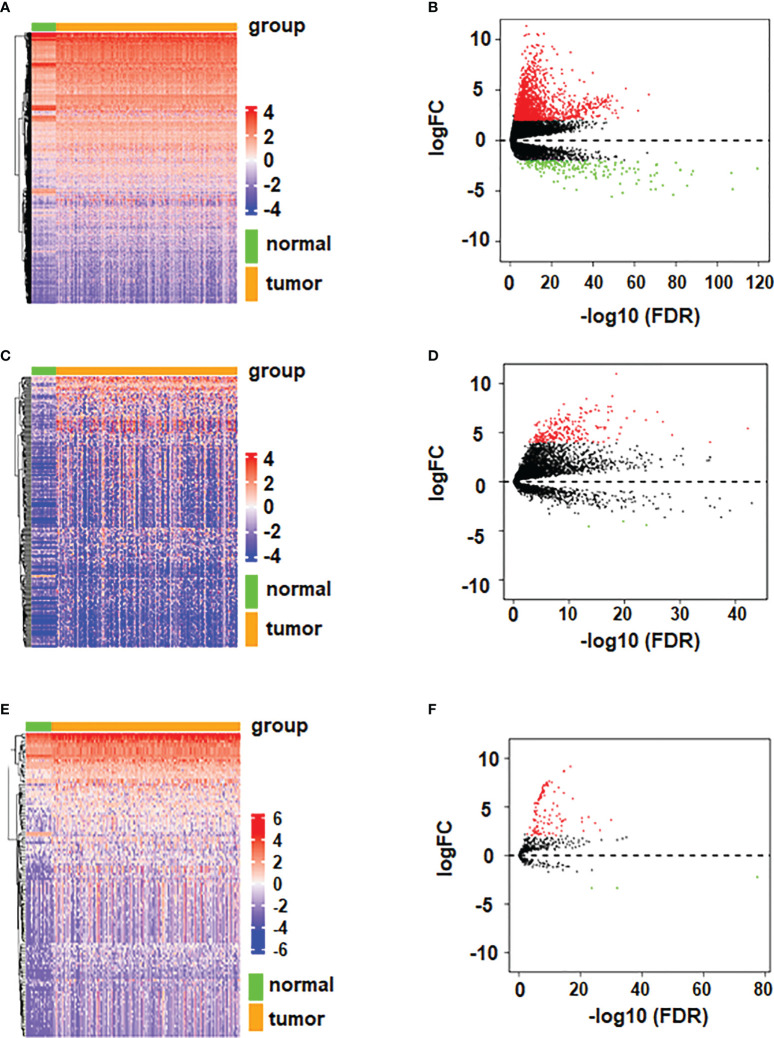
Volcano plots and heatmaps of differentially expressed mRNA, miRNA, and lncRNA. **(A)** Volcano plot of differentially expressed mRNA. **(B)** Heatmap of differentially expressed mRNA. **(C)** Volcano plot of differentially expressed lncRNA. **(D)** Heatmap of differentially expressed lncRNA. **(E)** Volcano plot of differentially expressed miRNA. **(F)** Heatmap of differentially expressed miRNA.

In order to better understand the expression of RNAs in HCC, we conducted differential analysis on mRNAs, lncRNAs and miRNAs, and the results are shown in **Figures 1A–F**. The Volcano diagram and Heatmap demonstrated that 1980 DEmRNAs had 1770 up-regulated and 210 down-regulated mRNAs (**Figures 1A, B**). There were 245 DElncRNAs, including 242 up-regulated and 3 down-regulated lncRNAs (**Figures 1C, D**). Moreover, 126 DEmiRNAs had 123 up-regulated and 3 down-regulated miRNAs (**Figures 1E, F**).

### Go and KEGG pathway analysis

**Figure 2:**
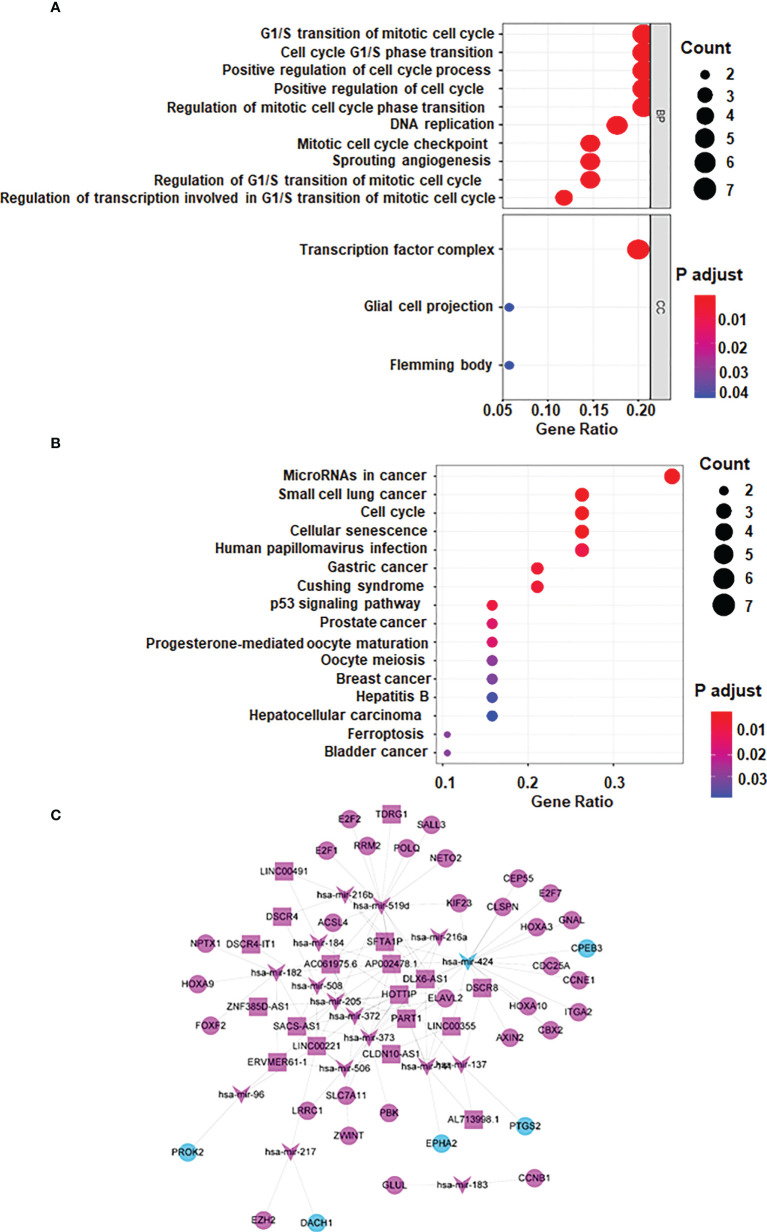
lncRNA-miRNA-mRNA ceRNA network. **(A)** GO analysis **(B)** KEGG Pathway Analysis **(C)** lncRNA-miRNA-mRNA ceRNA network generated by Cytoscape (version 3.6.1, https://cytoscape.org/). Red: upregulated RNAs. Green: downregulated RNAs. Triangle: miRNA. Diamond: lncRNA. Circular: mRNA.

The biofunctional analysis of the ceRNA co-modules is undertaken to further investigate the functional role of mRNAs/miRNAs/lncRNAs in carcinogenesis and if the ceRNA co-modules identified in the study are connected with HCC. Function analysis showed that mRNAs in ceRNA network were significantly enriched in G1/S transition of mototiv cell cycle, positive regulation of cell cycle process, hepatocellular carcinoma, and cancer related pathways (**Figures 2A, B**).

### Construction of a lncRNA-miRNA-mRNA ceRNA network

Based on the several databases, including miRcode database, a ceRNA network was constructed by differentially expressed lncRNAs, miRNAs and mRNAs. 8 DElncRNAs, 11 DEmiRNAs, 98 DEmRNAs are shown as nodes, and 156 interactions are shown as edges. Visualization is achieved by Cytoscape (**Figure 2C**).

### Prognosis model of hepatocellular carcinoma

**Figure 3:**
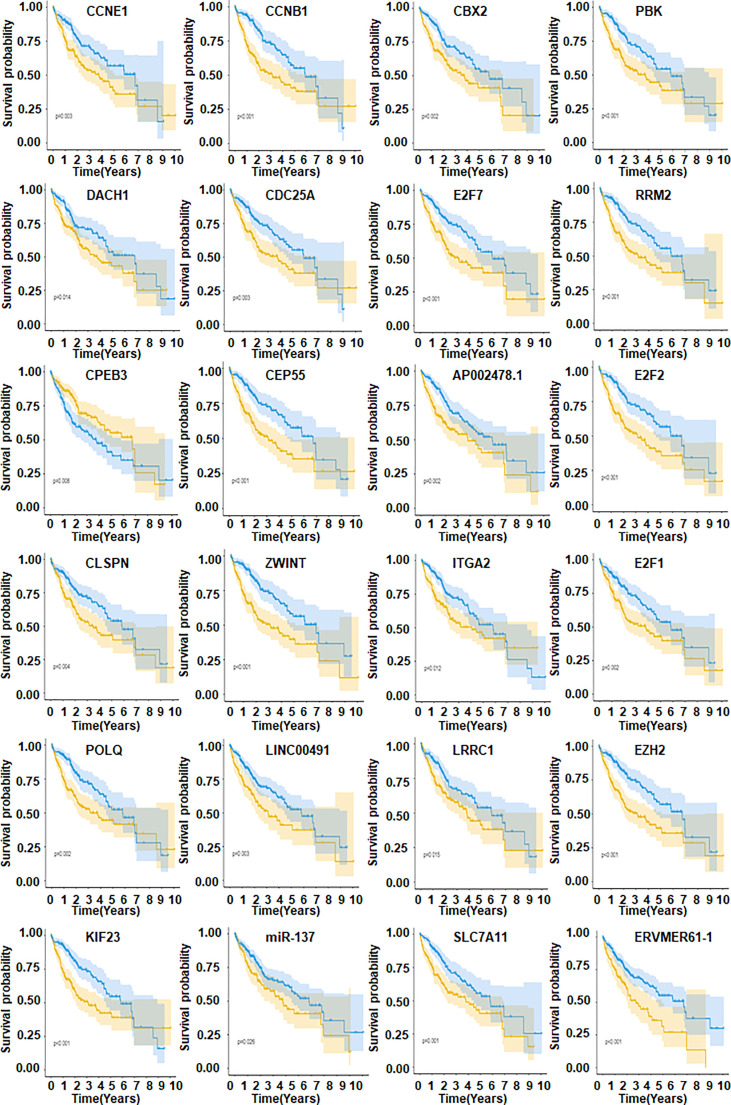
Kaplan-Meier method to find 24 genes.

**Figure 4:**
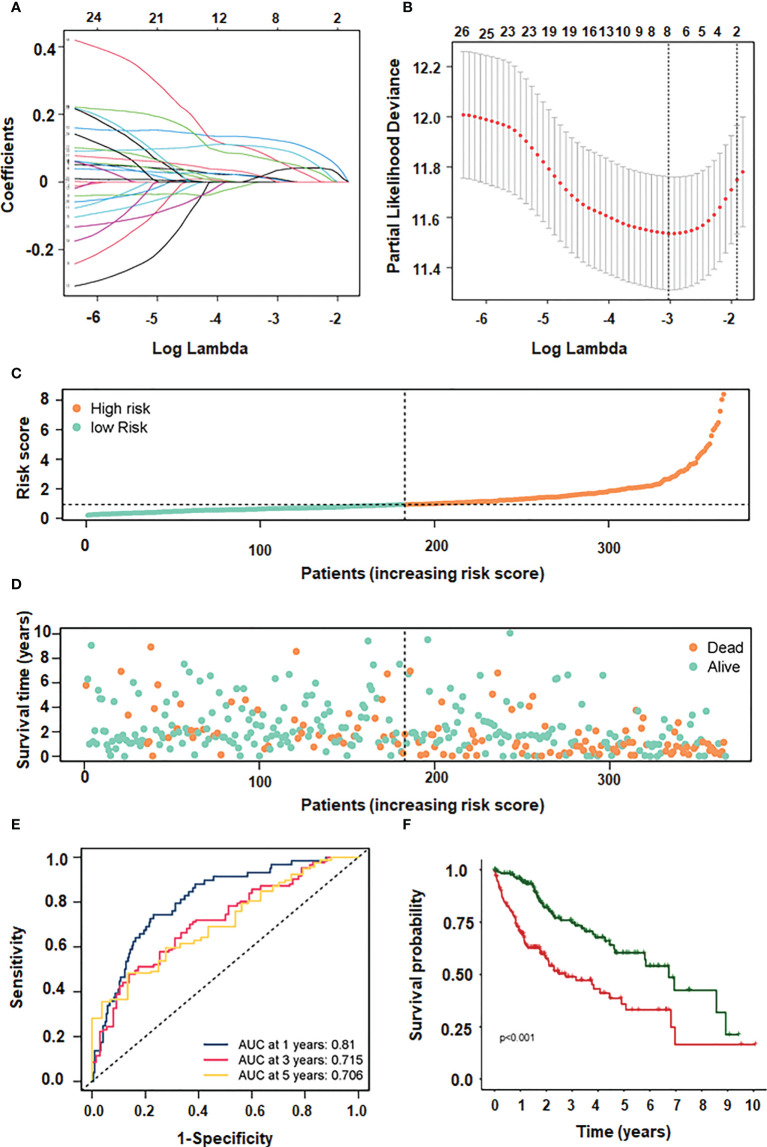
LASSO and multivariate regression models screening 4 genes. **(A)** different characteristics of genes and their corresponding coefficients. **(B)** cross-validation to build the model. **(C)** risk score with survival status cases. **(D)** risk score with survival time. **(E)** ROC curve plotted under the prediction model. **(F)** KM curve of this prediction model.

Our data imply that 24 mRNAs may be protective factors (**Figure 3**). We screened 24 genes associated with HCC prognosis (**Figure 3**). The prognosis of 31 genes was determined by univariate Cox regression analysis. Then, the results of Lasso regression showed that ERVMER61-1, SLC7A11, CEP55, CBX2, EZH2, PBK, AP002478.1 and miR-137 were associated with the HCC prognosis (*P <*0.05) (**Figure 4A**). The median risk score was used to categorise patients into high-risk and low-risk groups. The high-risk samples had a lower survival duration than the low-risk samples (**Figures 4B–D**). The AUC at 1 years, 3 years, 5 years were 0.764,0.61 and 0.802 (**Figure 4E**). The low-risk group had a worse chance of survival than the high-risk group (**Figure 4F**).

**Figure 5:**
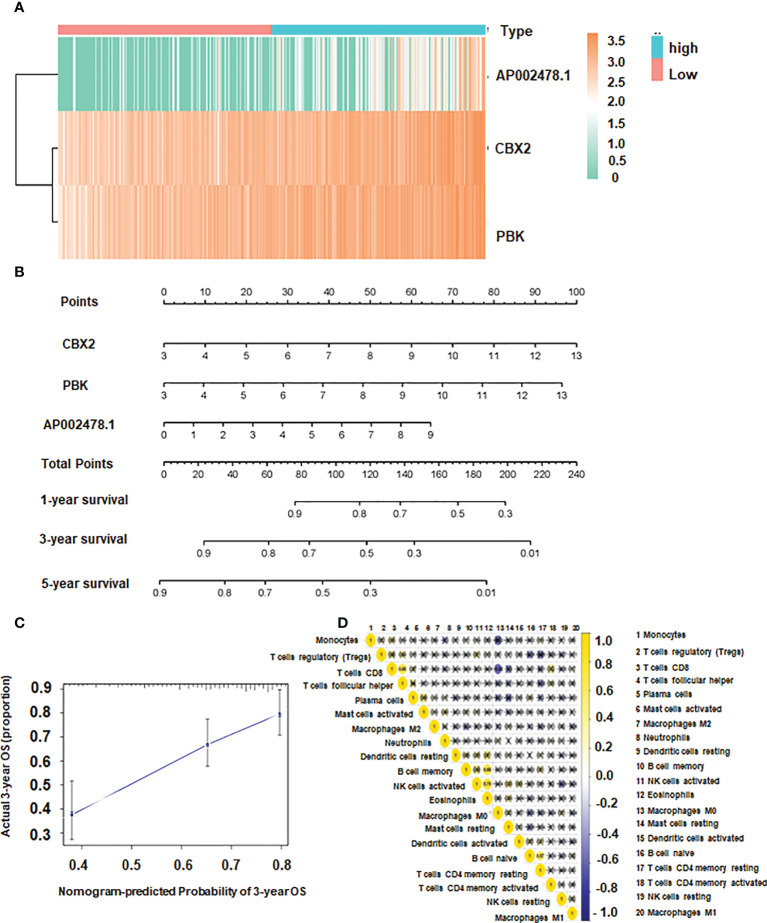
The association of CBX2, PBK, AP002478.1 and survival. **(A)** Heatmap of differentially expressed genes. **(B)** Nomogram based on multiple Cox regression. **(C)** Calibration curve for 3-year survival. The closer to the diagonal, the better the prediction effect. **(D)** Co-expression patterns among fractions of immune cells.

The independent prognostic indicators were then used to create the Nomogram, which forecasts survival rates for the first, three to five years. There is a nomogram model made up of CBX2, PBK and AP002478.1 (**Figures 5A, B**). The nomogram model’s calibration curves demonstrated good agreement between the anticipated 1/3/5-year overall survival rate and the actual survival rate, indicating that the model was accurate in its predictions (**Figure 5C**).

### Immune infiltration in HCC

**Figure 6:**
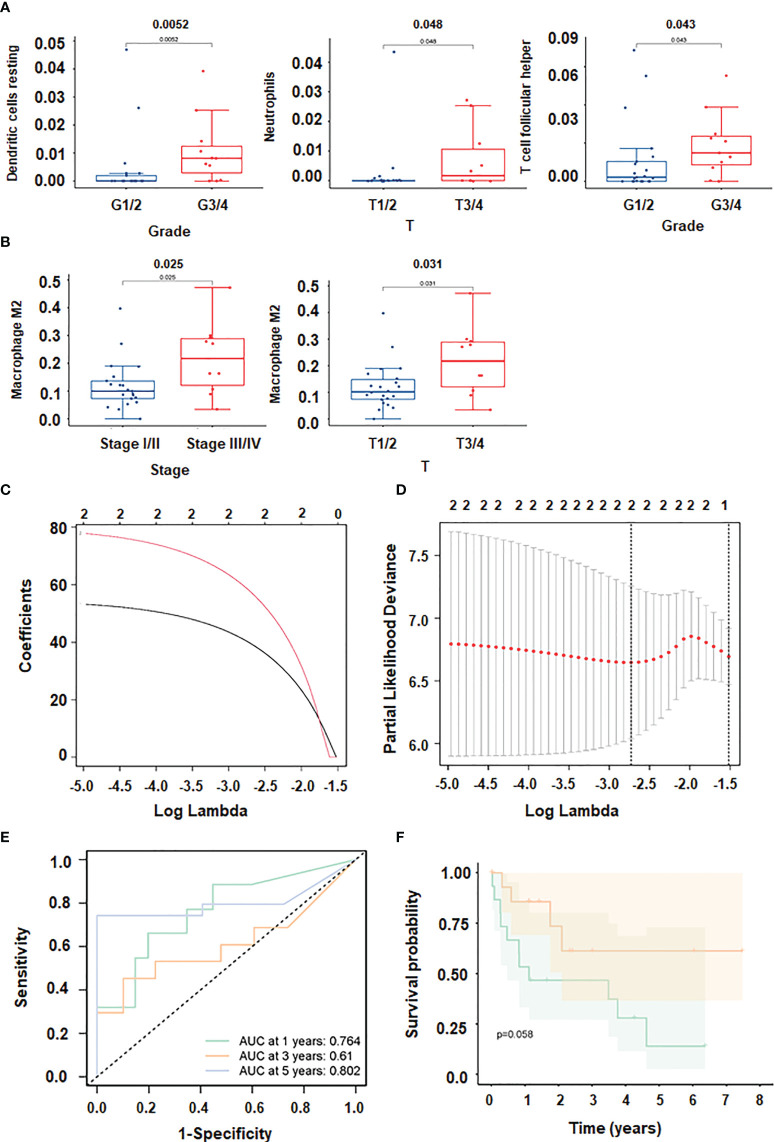
Immune cell infiltration in HCC. **(A, B)** Correlation between immune cells and clinical shape. **(C, D)**: different characteristics of immune cells and their corresponding coefficients; **(E)** ROC curve plotted under the prediction model). **(F)** KM curve of this prediction model.

CD8 T cells were positively correlated with T follicular helper cells, and the correlation coefficient was 0.65. Additionally, there was a positive link between Eosinophils and NK activated cells and B memory cells with a correlation coefficient of 0.75 and 0.68, respectively (**Figure 5D).** The expression of dendritic resting cells was higher in G3/4 and lower in G1/2 (**Figure 6A**). Similarly, T cell expression was higher in the G3/4 than in the G1/2 (**Figure 6A**). Neutrophils was highly expressed in T3/4 (**Figure 6A**). Macrophage M2 was highly expressed in T3/4 and stage III/IV (**Figure 6B**).

**Figure 7:**
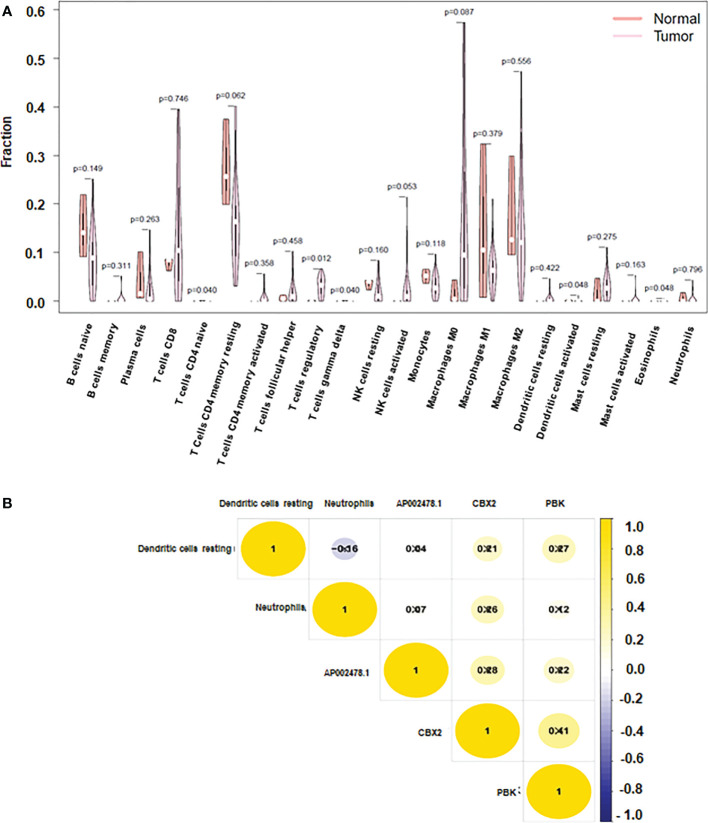
Immune cells and CBX2, PBK, AP002478.1 in HCC. **(A)** Heatmap of the three immune cells in the Cox regression model. **(B)** correlation between immune cells and genes.

To prevent overfitting and resolve severe collinearity, we performed lasso regression (**Figures 6C, D**). The AUC curve demonstrates that the nomogram survival prediction model had a high accuracy (**Figure 6E**). The AUC at 1 year, 3 years, and 5 years were 0.764, 0.61, and 0.812, respectively (**Figure 6E**). The high-risk group had worse survival than the low-risk group (**Figure 6F**). **Figure 7A** depicted that dendritic activated cells, and eosinophil were highly expressed in tumor cells. The Pearson correlation test was used to investigate the relationship between major genes in the ceRNA network and prognosis-related immune cells (**Figure 7B**).

### LncRNA HOTTIP promotes cell viability *via* targeting miR-205

**Figure 8:**
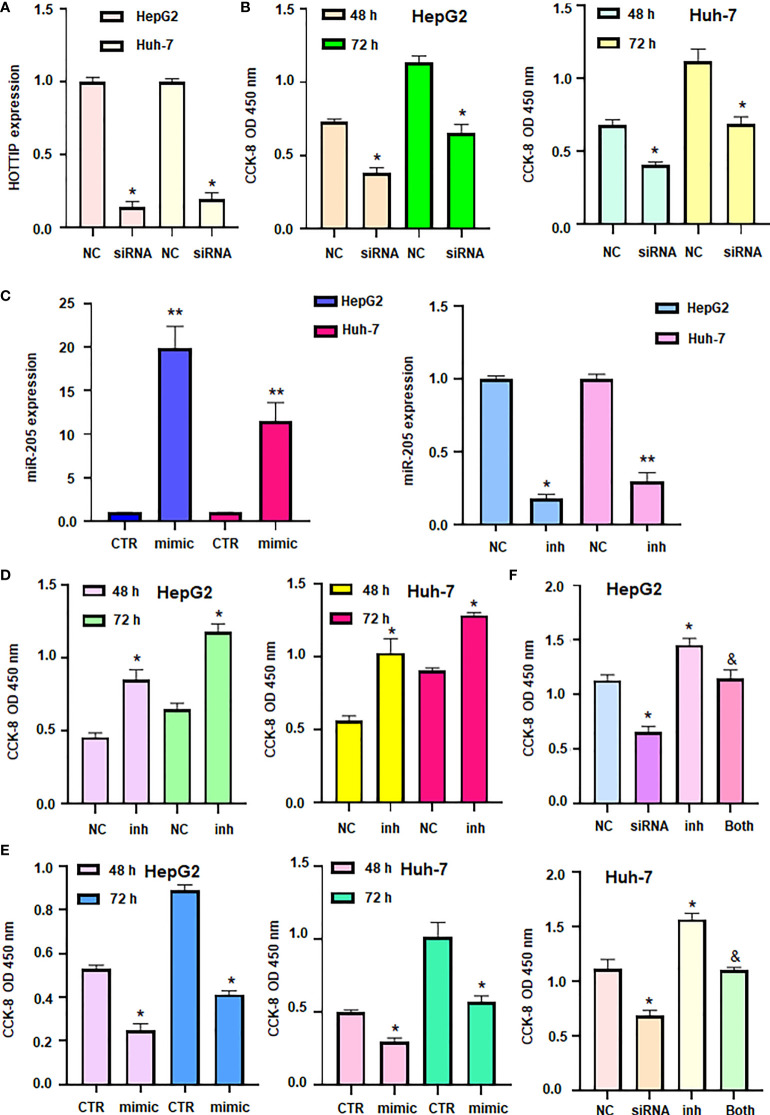
LncRNA HOTTIP promoted cell viability via miR-205 in HCC. **(A)** RT-PCR was done to measure the expression of HOTTIP in HCC cells after HOTTIP siRNA transfection. **(B)** CCK8 assay was done to measure cell viability in HCC cells after HOTTIP siRNA transfection. **(C)**: RT-PCR was done to measure the expression of miR-205 in HCC cells after miR-205 inhibitor or mimics transfection. **(D, E)** CCK8 assay was done to measure cell viability in HCC cells after miR-205 inhibitor or mimics transfection. **(F)** CCK8 assay was done to measure cell viability in HCC cells after HOTTIP siRNA transfection or miR-205 inhibitor or combination treatments. NC, negative control; inh, inhibitor; CTR, control. *P < 0.05; **P < 0.01 compared with NC or CTR; &P<0.05 compared with siRNA alone or inhibitor alone.

Our ceRNA network showed the role of lncRNA HOTTIP and miR-205 in HCC progression. To validate the function of lncRNA HOTTIP on cell viability of HCC, HOTTIP was downregulated by siRNA transfection in HCC cells (**Figure 8A**). CCK-8 data showed that downregulation of HOTTIP led to inhibition of cell viability in HepG2 and Huh7 cells (**Figure 8B**). Moreover, miR-205 expression level was increased in HCC cells after miR-205 mimic transfection, while miR-205 was downregulated in HCC cells after miR-205 inhibitor treatment (**Figure 8C**). Furthermore, inhibition of miR-205 increased viability of HCC cells, whereas upregulation of miR-205 decreased viability of HCC cells (**Figures 8D, E**). Notably, inhibition of miR-205 rescued HOTTIP siRNA-mediated suppression of cell viability in HCC (**Figure 8F**). Altogether, TOTTIP enhanced cell viability *via* targeting miR-205 in HCC cells.

## Discussion

HCC is one of the most common malignant tumors in the gastrointestinal system (38). At present, the molecular pathogenesis of HCC is still unclear (39). Therefore, it is very important to understand the molecular mechanism and process of HCC and identify new therapeutic targets to improve the clinical prognosis of patients. LncRNAs have been considered as an underappreciated novel therapeutic target (40). LncRNAs play important roles in gene regulation, including regulation of gene activation and silencing, X chromosome inactivation, alternative splicing, and post-translational regulation (41). Besides, lncRNAs have also been shown to be associated with tumor progression (12). Therefore, in this study, we studied the relationship between lncRNAs and liver cancer, and constructed a ceRNA regulatory network based on lncRNAs.

The current work used lncRNA-associated ceRNA to identify key biomarkers related to the prognosis of HCC. The lncRNAs LINC00491, DSCR4, AC061975.6, LINC00221, TDRG1 and HOTTIP served as hub nodes in the ceRNA network, which targeted other miRNAs and mRNAs. The ceRNA modulatory network of CBX2, PBK and AP002478.1 may impact HCC progression, according to KM survival and correlation study. Consistent with our study, one recent study suggested that LINC00491 promotes tumor growth and lung metastasis in mouse xenografts. LINC00491 is highly expressed in HCC patients and is associated with poor prognosis. More deeply, LINC00491 promotes HCC progression by sponging miR-324-5p/ROCK1 and may be a potential therapeutic target for HCC (42). In addition, overexpression of lncRNA TDRG1 promotes the viability, invasion and migration of endometrial cancer cells, inhibits cell apoptosis, and upregulates the expression of VEGF-A, PI3K, Bcl-2, MMP2 and surviving (43). Similarly, TDRG1 overexpression also promoted GC growth and metastasis *in vitro* and *in vivo*, which was regulated by the miR-873-5p/HDGF pathway (44).

LncRNA HOTTIP has been reported to regulate HCC development and progression (45). In our study, we found that HOTTIP can act as a node of ceRNA and is closely related to the development of HCC. Meanwhile, a study also demonstrated that HOTTIP acts as a molecular sponge for miR-148a-3p to increase WNT1 expression, thereby modulating the CSC-like properties of breast cancer, suggesting that HOTTIP is a new target for breast cancer treatment (46). LncRNA HOTTIP expression was correlated with tumor recurrence in HCC patients following liver transplantation (47). Similarly, lncRNA HOTTIP/HOXA13 expression was linked to tumor progression and therapeutic outcome in HCC patients (19). Additionally, lncRNA HOTTIP was elevated in HCC and was regulated by miR-125b (48). Ge et al. found that miR-192 and miR-204 reduced the expression of lncRNA HOTTIP and impaired GLS1-involved glutaminolysis in HCC (49). Tang et al. reported that solamargine inhibited the expression of lncRNA HOTTIP and TUG1, increased miR-4726-5p expression and inhibited MUC1 expression in HCC cells, leading to inhibition of cell growth and promotion of sorafenib efficacy (50). Our *in vitro* data further identified that HOTTIP promoted viability of HCC cells *via* regulation of miR-205. Dong et al. discovered that lncRNA HOTTIP promoted cisplatin resistance *via* targeting miR-205 and modulating ZEB2 in ovarian cancer cells (51). It is pivotal to determine whether lncRNA HOTTIP enhanced viability of HCC cells *via* regulation of miR-205/ZEB2 axis.

In our study, we found that dendritic activated cells and eosinophil are closely related to liver cancer. Dendritic cells are the most powerful professional antigen-presenting cells, widely distributed in peripheral lymphoid tissues, and play an important role in the immune regulation of the body. Liver DCs play an important role in the regulation of hepatic immune responses, and their precursor cells can induce antigen-specific immune tolerance in T cells that have not been stimulated by specific antigens, while for T memory cells, they are strong immune stimulators (52–54). *In vitro* studies have shown that once hepatic DC precursor cells are induced by antigen, they can also stimulate the proliferation of non-antigen-sensitized allogeneic T cells (55). There are some limitations in our study. First of all, the main disadvantage of this study is the lack of *in vivo* validation. Secondly, this study only included the TCGA database, and more databases should be included for research in the future.

## Conclusion

The TCGA database’s analysis of the lncRNA, miRNA, and mRNA expression profiles of HCC resulted in the creation of a ceRNA network. It was discovered that lncRNAs may play a role in carcinogenesis and may help predict the overall survival of patients with HCC. To ascertain their viability as therapeutic or diagnostic biomarkers, it is important to investigate the probable processes of these lncRNAs in HCC. However, further research on the clinical practice of this nomogram combined model and the exact molecular mechanism of the two lncRNAs identified in this study are required.

## Data availability statement

The original contributions presented in the study are included in the article/supplementary material. Further inquiries can be directed to the corresponding authors.

## Author contributions

WC, FC, MG, ZJ and LS performed the experiments, analyzed the data. WC and JW wrote the manuscript. Z-WW edited the manuscript. JW supervised this study. All authors contributed to the article and approved the submitted version.

## Conflict of interest

The authors declare that the research was conducted in the absence of any commercial or financial relationships that could be construed as a potential conflict of interest.

## Publisher’s note

All claims expressed in this article are solely those of the authors and do not necessarily represent those of their affiliated organizations, or those of the publisher, the editors and the reviewers. Any product that may be evaluated in this article, or claim that may be made by its manufacturer, is not guaranteed or endorsed by the publisher.

## Abbreviations

ceRNA, competing endogenous RNA; circRNA, circular RNAs; CSC, cancer stem cells; DSCR4, Down Syndrome critical region 4; EZH2, Enhancer of Zeste 2; GO, gene ontology; HCC, hepatocellular carcinoma; HOTTIP, HOXA distal transcript antisense RNA; HOXA13, homeobox A13; KEGG, The Kyoto Encyclopedia of Genes and Genomes; lncRNA, long noncoding RNA; MEG3, maternally expressed 3; miRNA, microRNA; mRNA, messenger RNA; SLC7A11, solute carrier family 7 member 11; TCGA, The Cancer Genome Atlas; TDRG1, testis development related 1; UCA1, urothelial cancer associated 1.
